# Efficacy of reinforcing sutures for prevention of anastomotic leakage after low anterior resection for rectal cancer: A systematic review and meta‐analysis

**DOI:** 10.1002/cnr2.1941

**Published:** 2024-01-04

**Authors:** Shuanhu Wang, Yi Zhang, Song Tao, Yakui Liu, Yi Shi, Jiajia Guan, Mulin Liu

**Affiliations:** ^1^ Department of Gastrointestinal surgery The First Affiliated Hospital of Bengbu Medical College Bengbu Anhui China

**Keywords:** anastomotic leakage, rectal cancer, sutures

## Abstract

**Background and Objectives:**

Anastomotic leakage is a serious complication following surgery for cancer of the rectum. It is not clear whether reinforcing sutures could prevent anastomotic leakage. Therefore, this study aims at evaluating the efficacy of reinforcing sutures on anastomotic leakage.

**Methods:**

We searched PubMed, Embase, and the Cochrane Library databases from inception to January 31, 2023. We included studies comparing anastomosis with reinforcing sutures to anastomosis without reinforcing sutures after low anterior resection. Risk of bias was assessed by the Cochrane tool for RCTs and the Risk of Bias in Non‐Randomized Studies (ROBINS)‐I tool for observational studies. The overall quality of evidence for primary outcome was assessed using Grading of Recommendations Assessment, Development, and Evaluations methodology.

**Results:**

Two RCTs (345 patients) and four observational studies (783 patients) were included. Anastomotic leakage occurred in 4.4% (24 of 548) of patients with reinforcing sutures and 11.9% (69 of 580) of patients without reinforcing sutures. Meta‐analysis showed a lower incidence of anastomotic leakage (RR, 0.41; 95% CI 0.25 to 0.66, low certainty) in patients with reinforcing sutures. Operative time (WMD, −3.66; 95% CI −18.58 to 11.25) and reoperation for anastomotic leakage (RR, 0.69; 95% CI 0.23 to 2.08) were similar between patients with reinforcing sutures and those without reinforcing sutures.

**Conclusions:**

While observational data suggest that, there is a clear benefit in terms of reducing the risk of anastomotic leakage with the use of reinforcing sutures, RCT data are less clear. Further large, prospective studies are warranted to determine whether a true clinically important benefit exists with this technique.

## 1 INTRODUCTION

Rectal cancer is the eighth most common malignancy worldwide.^1^ With the widespread use of the circular stapler and the double stapler techniques, more anus is preserved in an increasing number of patients. However, anastomotic leakage may also come with anal preservation surgery. The incidence of anastomotic leakage varies from 3.4% to 20%.^2^, ^3^ Anastomotic leakage not only decreases the patients' quality of life,^4^ but also negatively affects their survival.^5^, ^6^

Several methods are used for preventing anastomotic leakage after low anterior resection in patients with rectal cancer, such as defunctioning loop ileostomy,^7^ three‐row circular staplers,^8^ low inferior mesenteric artery (IMA) ligation,^9^ and transanal drainage tube use.^10^ All of these methods have problems of one kind or another, such as defunctioning loop ileostomy requiring secondary surgery, patients with transanal drainage tube placement experience perianal pain. If there is a technology that can reduce the incidence of anastomotic leakage, it will reduce the short and long‐term harm caused by anastomotic leakage. The technique is reinforcing sutures. While one study has found that the use of reinforcing sutures in anal preservation surgery significantly reduces the incidence of anastomotic leakage,^11^ another has not.^12^ Therefore, we conducted this study to summarize the efficacy of reinforcing sutures for the prevention of anastomotic leakage in patients with rectal cancer after low anterior rectal resection.

## 2 MATERIALS AND METHODS

### 2.1 Literature search and inclusion criteria

Two authors (S.W. and Y.Z.) searched electronic databases (PubMed, Embase, and the Cochrane Library) from inception to January 31, 2023. Search terms included “rectal cancer,” “reinforcing sutures,” and “anastomotic leakage” (Electronic Supplementary Material: Search Strategy).

The inclusion criteria for this study were as follows: (1) population: patients receiving low anterior resection to treat cancer of the rectum; (2) intervention: reinforcing sutures at the anastomosis; (3) comparison: no reinforcing sutures at the anastomosis; (4) outcome measure: the incidence of anastomotic leakage; (5) study design: all types of study.

The exclusion criteria were as follows: (1) Articles with no data available; (2) Articles withdrawn.

### 2.2 Data extraction and quality assessment

Two authors (S.W. and S.T.) independently screened the titles and abstracts of the articles identified by the search strategy and extracted the data. Disagreements were resolved through discussion. The following study variables were extracted from each selected study: first author, publication year, publishing country, published journal, study design, intervention description and characteristics. The primary outcome was the incidence of anastomotic leakage. Secondary outcomes included operative time and reoperation for anastomotic leakage. The following were considered anastomotic leakage: discharge of gas or feces through the pelvic drain, and fluid/air bubbles surrounding the anastomosis on computed tomography (CT).

Risk of bias of RCTs was assessed by the Cochrane tool for RCTs by two authors (S.W. and Y.L.). Risk of bias was rated as “low risk,” “unclear risk,” and “high risk” according to seven domains.^13^ Risk of bias of observational studies was assessed by the Risk of Bias in Non‐Randomized Studies (ROBINS)‐I tool.^14^ Risk‐of‐bias VISualization (robvis) was used to create risk‐of‐bias plots.^15^

### 2.3 Statistical analysis

We used Review Manager software (Version 5.4, Copenhagen, Denmark) for statistical analysis. We presented results as pooled risk ratios (RR) or pooled weighted mean difference (WMD) along with 95% confidence intervals (CIs). We assessed heterogeneity using *χ* ^2^ and *I* ^2^ statistics. Heterogeneity was considered significant if the *P* value (χ^2^) was <.1 and *I* ^2^ was >50%. We used a random‐effects model without considering heterogeneity. A priori subgroup analyses were not specified. Sensitivity analysis was performed by omitting one study at a time to assess the impact of each study on the overall risk assessment. A *P* value <.05 for the overall effect test was considered significant. There was no assessment of publication bias because the number of studies in the analysis was less than 10.^16^

### 2.4 Certainty of evidence

The overall quality of the evidence for the primary outcome was evaluated using the Grading of Recommendations Assessment, Development and Evaluation (GRADE) approach.^17^ The GRADE approach specifies four levels of the certainty for a body of evidence for a given outcome: high, moderate, low, and very low. We created a GRADE evidence profile using the guideline development tool (gradepro.org).

## 3 RESULTS

### 3.1 Search results and study characteristics

**FIGURE 1:**
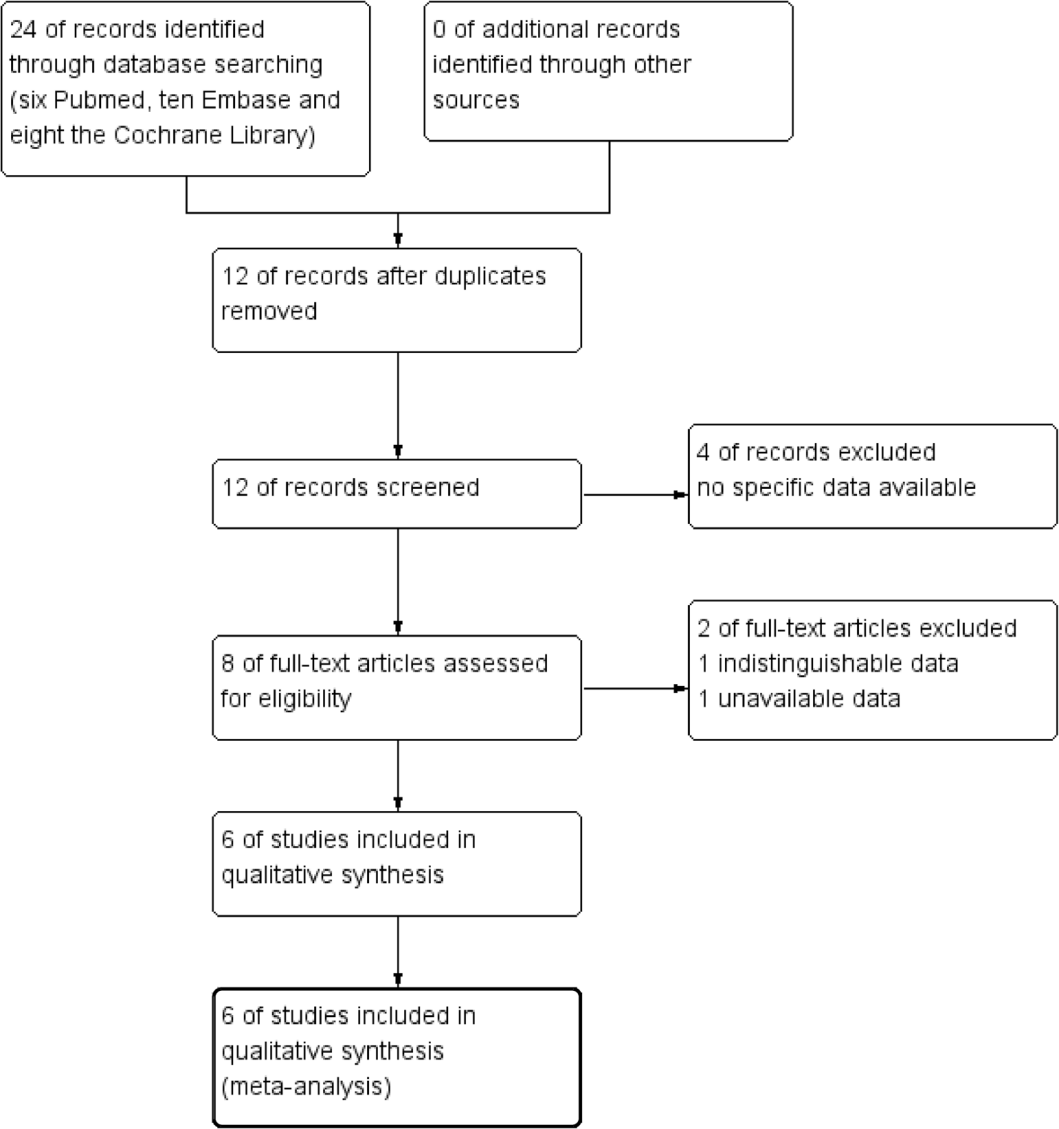
Process diagram of the literature screening and selection.

The initial search identified 24 studies (six Pubmed, ten Embase and eight the Cochrane Library). After removing 12 duplicate studies, 12 articles remained. After reading the titles and abstracts, four studies were excluded due to irrelevant content, leaving eight articles for full‐text review. After further review, two studies were excluded for the following reasons: one study only reported methodology and did not include specific data; the other study included other diseases, and we could not extract data related to low anterior resection for rectal cancer. Finally, this meta‐analysis included six studies (Figure 1).The included studies were published between 2013 and 2022. The sample sizes of the studies ranged from 54 to 319 patients. There were four observational studies^11^, ^18^, ^19^, ^20^ and two randomized controlled trials,^21^, ^22^ which included 1128 patients. A total of 548 patients received reinforcing sutures and 580 did not. Of the six studies, all reported the incidence of anastomotic leakage, three reported operation time, and three reported reoperations for anastomotic leakage. Further characteristics of these studies are shown in Tables 1 and 2.

**TABLE 1:** Details of the included studies.

Reference	Year	Country	Journal	Study design	Tumor location	Sutures	Definition of anastomotic leakage	Surgical approach
Hashida et al.^11^	2022	Japan	Wideochir Inne Tech Maloinwazyjne	Cohort study	<10 cm from the anal verge	3–0 PDS	Radiologic evidence	Transabdominal
Baek et al.^18^	2013	South Korea	World J Gastroenterol	Cohort study	<10 cm from the anal verge	Sutures (unspecified)	Clinical or radiologic evidence	Transanal
Ban et al.^19^	2022	China	World J Gastrointest Surg	Cohort study	<10 cm from the anal verge	3–0 V‐Loc 180 sutures	Clinical or radiologic evidence	Transabdominal
Maeda et al.^20^	2015	Japan	Surg Endosc	Cohort study	within 15 cm from the anal verge	4–0 PDS	Clinical or radiologic evidence	Transabdominal
Altomare et al.^21^	2021	Italy	Colorectal Dis	RCT (Multi‐Center)	8–3 cm from the dentate line	3–0 Vicryl	Clinical or radiologic evidence	Transanal
He et al.^22^	2018	China	Zhonghua Wei Chang Wai Ke Za Zhi	RCT (Single center)	within 15 cm from the anal verge	4–0 absorbable sutures	Clinical or radiologic evidence	Transabdominal

Abbreviations: PDS, Polydioxanone synthetic absorbable suture; RCT, Randomized controlled trial.

**TABLE 2:** Characteristics of the included studies.

Reference	Approach	Sample size	Neoadjuvant chemoradiotherapy	Diverting ileostomy	Anastomotic leakage	Operative time, min (mean ± SD)	Reoperation for anastomotic leakage
Hashida et al.^11^	Reinforcing sutures	72	0	0	1	NA	NA
	Non‐reinforcing sutures	81	0	0	10	NA	NA
Baek et al.^18^	Reinforcing sutures	47	0	6	3	198.3 ± 75.7	2
	Non‐reinforcing sutures	63	0	19	5	212.1 ± 65.0	2
Ban et al.^19^	Reinforcing sutures	168	0	0	8	150.4 ± 25.1	2
	Non‐reinforcing sutures	151	0	0	17	146.6 ± 20.2	13
Maeda et al.^20^	Reinforcing sutures	91	0	0	3	NA	NA
	Non‐reinforcing sutures	110	0	0	15	NA	NA
Altomare et al.^21^	Reinforcing sutures	25	12	0	4	161.20 ± 68.68	1
	Non‐reinforcing sutures	29	9	29	5	181.11 ± 51.69	2
He et al.^22^	Reinforcing sutures	145	0	0	5	NA	NA
	Non‐reinforcing sutures	146	0	0	17	NA	NA

Abbreviations: NA, Not applicable; SD, Standard deviation.

### 3.2 Risk of bias

The risk of bias for the two RCTs was assessed using the Cochrane tool, and the allocation concealment was unclear for both studies.^21^, ^22^ The risk of bias for the four observational studies was assessed using ROBINS‐I tool. Three studies^11^, ^19^, ^20^ were considered to be at moderate risk of overall bias, and one study^18^ was considered to be at serious risk of overall bias (Electronic Supplementary Material: Details of the risk of bias).

### 3.3 Primary outcome: incidence of anastomotic leakage

**FIGURE 2:**
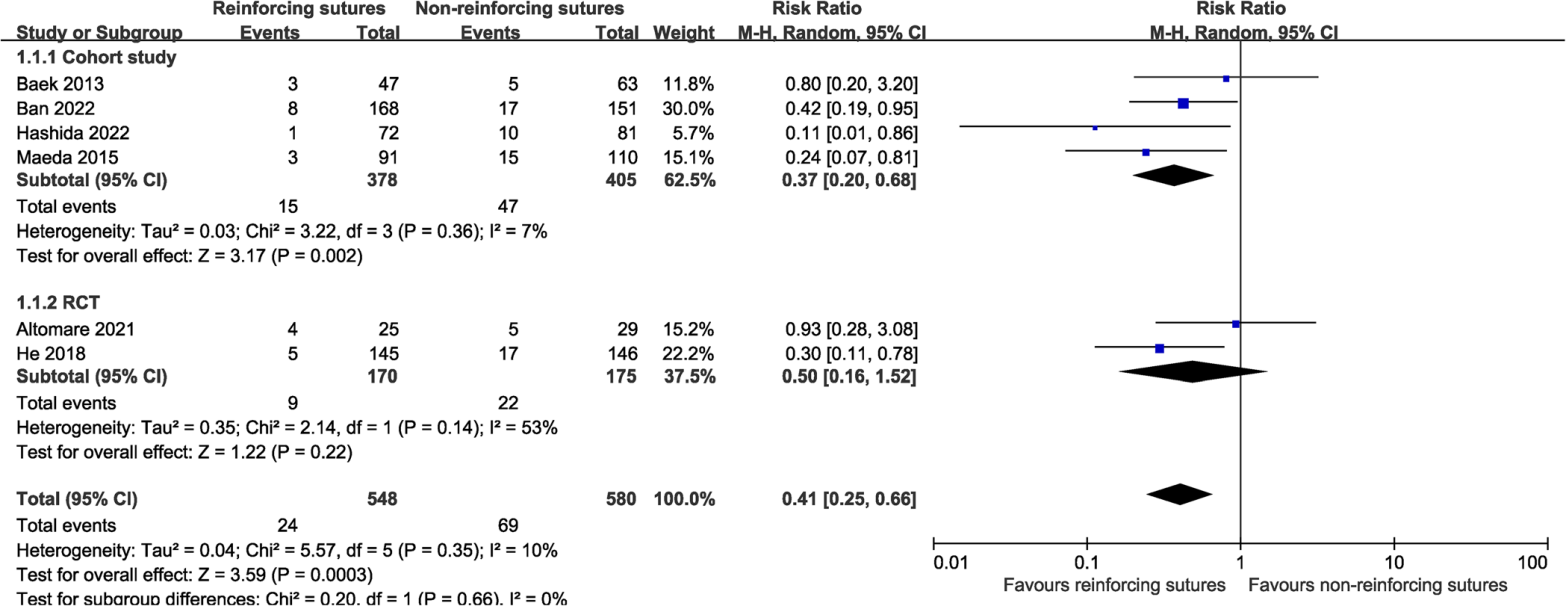
Forest plot of the incidence of anastomotic leakage.

In one of the six studies, anastomotic leakage was diagnosed based solely on radiologic evidence, while in the other studies the diagnosis of anastomotic leakage was based on clinical and radiologic evidence. The incidence of anastomotic leakage in the group with reinforcing sutures and that without reinforcing sutures was 4.4% and 11.9%, respectively. Meta‐analysis showed a lower incidence of anastomotic leakage (RR, 0.41; 95% CI 0.25 to 0.66) in patients with reinforcing sutures. Forest plots are shown in Figure 2. GRADE assessments are shown in Supplement (Electronic Supplementary Material: GRADE assessments – anastomotic leakage).

### 3.4 Subgroup analysis

**FIGURE 3:**
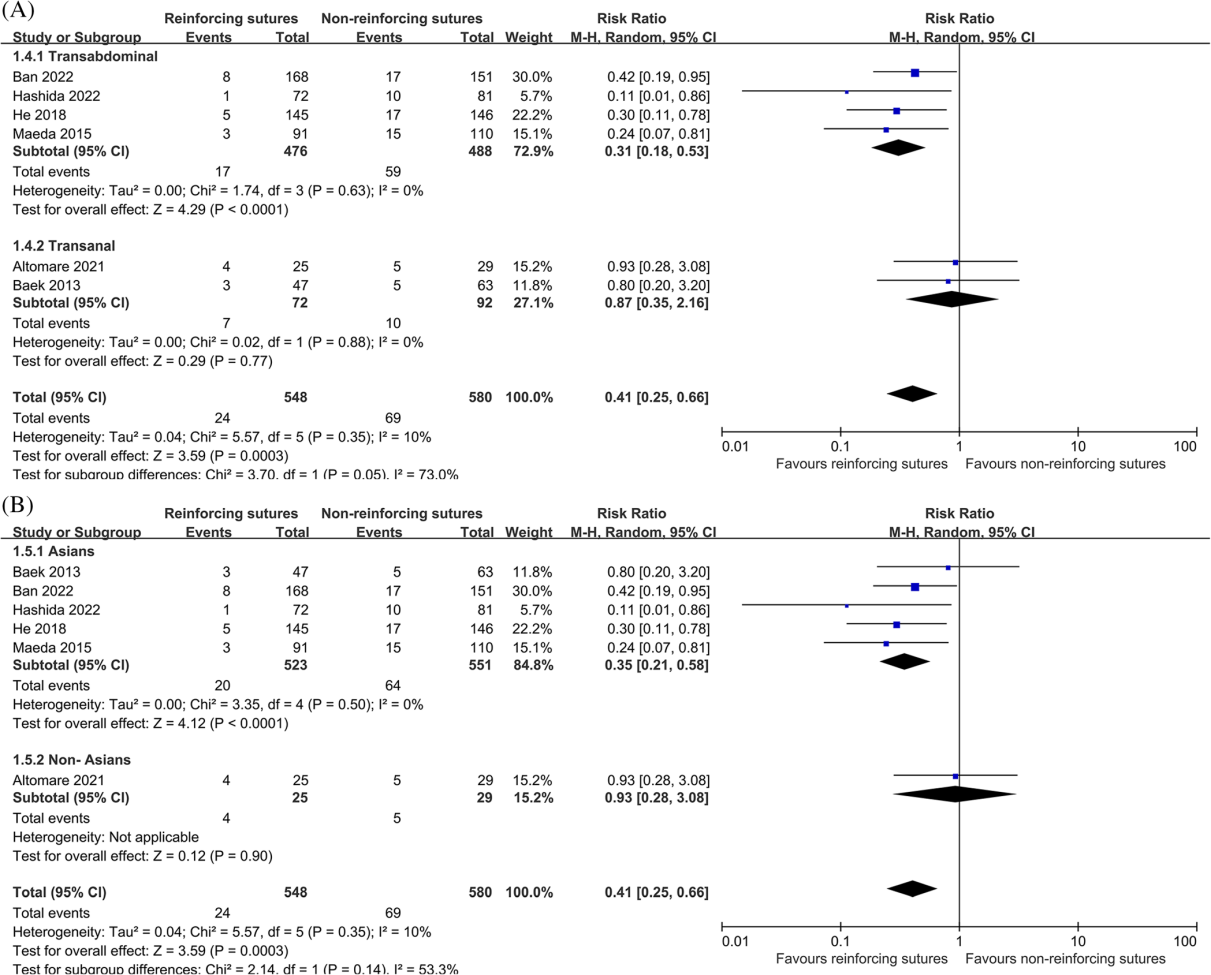
Forest plot of subgroup analysis.

Although no significant heterogeneity was found in the six studies (*P* = .35, *I* ^2^ = 10%, Figure 2), we performed subgroup analyses to assess the effect of grouping factors on the outcomes. These subgroups were based on surgical approach (transabdominal vs. transanal) and geographic area of the patients (Asians vs. Non‐Asians) (Figure 3). The results indicated that there were no significant differences between the subgroups.

### 3.5 Sensitivity analysis

We performed sensitivity analysis by excluding one study at a time. The pooled RR for anastomotic leakage ranged from 0.35 (95% CI 0.21 to 0.58, *P* < .0001) to 0.44 (95% CI 0.24 to 0.81, *P* = .009) between the two groups. The direction and consistency of the results remained unchanged.

### 3.6 Secondary outcomes

**FIGURE 4:**
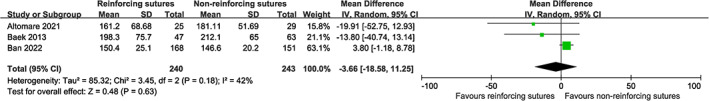
Forest plot of operative time.

Three studies reported operative time, with no heterogeneity (*P* = .18, *I* ^2^ = 42%). In the random‐effects model, operative time was similar between the group with reinforcing sutures and that without reinforcing sutures groups (WMD, −3.66; 95% CI −18.58 to 11.25) (Figure 4).

**FIGURE 5:**
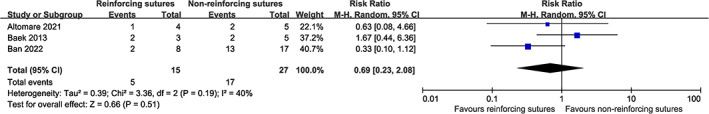
Forest plot of reoperation for anastomotic leakage.

Three studies reported reoperation for anastomotic leakage. The pooled results of these studies showed that reinforcing sutures were associated with a similar risk of reoperation for anastomotic leakage (RR, 0.69; 95% CI 0.23–2.08, Figure 5), with no heterogeneity among them (*P* = .19, *I* ^2^ = 40%).

## 4 DISCUSSION

In this study, we evaluated the efficacy of reinforcing sutures after low anterior resection for rectal cancer. The results revealed that reinforcing sutures reduced the incidence of anastomotic leakage while making no significant difference in terms of operative time and reoperation for anastomotic leakage.

This is the first systematic review and meta‐analysis on reinforcing sutures after rectal cancer surgery. A previous systematic review and meta‐analysis on reinforcing sutures mainly focused on the field of bariatric surgery.^23^, ^24^ Their results showed that staple line reinforcement during laparoscopic sleeve gastrectomy or gastric bypass reduced the incidence of anastomotic leakage. Leakage after bariatric surgery is very rare, less than 1%.^25^ The incidence of anastomotic leakage after rectal cancer surgery is more than 3%, even up to 20%.^2^, ^3^ Therefore, reinforcing sutures may be more important in rectal cancer surgery than in bariatric surgery.

To reduce the incidence of anastomotic leakage, a tension‐free anastomosis is beneficial.^26^ The double‐stapling technique has widely been used in rectal surgery. Two corners made by crossing the circular and linear staple lines are weak; thus, reinforcing sutures are placed at the crossing point of the staple lines. Reinforcing sutures can be used to stop bleeding, and they can reduce the tension at the anastomosis, thereby preventing the occurrence of anastomotic leakage. In addition to anastomotic tension, diverting ileostomy also has an impact on anastomotic leakage. Some studies have found that diverting ileostomy may reduce the incidence of anastomotic leakage.^7^, ^27^ In the articles included in our analysis, the number of temporary diverting ileostomies performed was significantly higher in the non‐reinforcing sutures group. This has likely reduced the incidence of anastomotic leakage in the non‐reinforcing sutures group. Even so, the results of our meta‐analysis showed that the reinforcing sutures group had a lower incidence of anastomotic leakage than the non‐reinforcing sutures group. This further illustrates the stability of the results.

The incidence of anastomotic leakage in transabdominal and transanal rectal cancer surgery remains controversial.^28^, ^29^ We performed subgroup analysis based on different surgical approach. In the transanal subgroup, there was no difference in the incidence of anastomotic leakage between reinforcing sutures and non‐reinforcing sutures. In the transabdominal subgroup, the incidence of anastomotic leakage in the reinforcing sutures group was significantly lower than that in the non‐reinforcing sutures group. Hence, it is more beneficial to implement reinforcing sutures in patients undergoing transabdominal approach. The results were similar in Asian and non‐Asian patients.

Although the results showed the effectiveness of reinforcing sutures, it was also worth paying attention to whether reinforcing sutures would increase the operative time or other risks. Our study found that reinforcing sutures did not significantly increase the operative time. Six to eight interrupted sutures or 3–0 barbed thread for continuous sutures were placed along the anastomosis. The use of barbed thread made suturing easier, and there were fewer intermittent sutures, so operative time was not increased. Once the anastomotic leakage occurred, some patients had to undergo a reoperation. Patients with reoperation for anastomotic leakage were further treated by Hartmann or ileostomy operations. The results showed that reinforcing sutures did not significantly increase the reoperation for anastomotic leakage.

This review builds on clear methodology, such as predefined inclusion criteria, outcome measures, study quality appraisal, and GRADE assessments a priori. The majority of the included studies had low or moderate risk of bias. Certainty assessment of the primary outcome was low. However, there are some limitations that should be considered. First, in the included studies, the distance between the tumor and the anal verge was less than 10 cm, and in some, it was less than 15 cm. The incidence of anastomotic leakage varies with the distance between the tumor and the anal verge.^30^, ^31^ Second, there are different ways to reinforce sutures, including interrupted suture and continuous suture, which may cause confusion. Finally, the large effect sizes were driven by observational data, which were at significant risk of bias from confiding.

## 5 CONCLUSIONS

While observational data suggest that, there is a clear benefit in terms of reducing the risk of anastomotic leakage with the use of reinforcing sutures, RCT data are less clear. Further large, prospective studies are warranted to determine whether a true clinically important benefit exists with this technique.

## AUTHOR CONTRIBUTIONS

Shuanhu Wang conceived the study, extracted and analyzed the data, and drafted the manuscript. Yi Zhang collected the data and helped to draft the manuscript. Tao Song extracted the data. Yakui Liu and Yi Shi analyzed the data. Jiajia Guan and Mulin Liu participated in the study design.

## CONFLICT OF INTEREST STATEMENT

The authors declare that they have no competing interests.

## Supporting information


**Supplementary Material**: Search Strategy.

**Supplementary Material**: Details of the risk of bias.

**Supplementary Material**: GRADE assessments – anastomotic leakage.

## Data Availability

Data sharing is not applicable to this article as no new data were created or analyzed in this study.
