# Supplementary material for: Efficacy of reinforcing sutures for prevention of anastomotic leakage after low anterior resection for rectal cancer: A systematic review and meta‐analysis
Source: Cancer Rep (Hoboken). 2024 Jan 4;7(2):e1941. doi: 10.1002/cnr2.1941 (PMC10849930; doi:10.1002/cnr2.1941)
Supplement: Supplementary file 1 — Supplementary Material: Search Strategy. [file CNR2-7-e1941-s001.docx]

**Search strategy in PubMed**

("Rectal Neoplasms"[MeSH Terms] OR ("neoplasm rectal"[Title/Abstract] OR "rectal neoplasm"[Title/Abstract] OR "rectum neoplasms"[Title/Abstract] OR "neoplasm rectum"[Title/Abstract] OR "rectum neoplasm"[Title/Abstract] OR "rectal tumors"[Title/Abstract] OR "rectal tumor"[Title/Abstract] OR "tumor rectal"[Title/Abstract] OR "neoplasms rectal"[Title/Abstract] OR "cancer of rectum"[Title/Abstract] OR "rectum cancers"[Title/Abstract] OR "rectal cancer"[Title/Abstract] OR "cancer rectal"[Title/Abstract] OR "rectal cancers"[Title/Abstract] OR "rectum cancer"[Title/Abstract] OR "cancer rectum"[Title/Abstract] OR "cancer of the rectum"[Title/Abstract])) AND ("reinforcing"[Title/Abstract] AND ("Suture"[Title/Abstract] OR "staple surgical"[Title/Abstract] OR "staples surgical"[Title/Abstract] OR "surgical staples"[Title/Abstract] OR "surgical staple"[Title/Abstract])) AND ("anastomotic leak"[MeSH Terms] OR ("anastomotic leaks"[Title/Abstract] OR "leak anastomotic"[Title/Abstract] OR "leaks anastomotic"[Title/Abstract] OR "anastomotic leakage"[Title/Abstract] OR "anastomotic leakages"[Title/Abstract] OR "leakage anastomotic"[Title/Abstract] OR "leakages anastomotic"[Title/Abstract]))

**Search strategy in Embase**

#1 'rectum cancer'/exp

#2 'cancer of the lower rectum':ab,ti

#3 'cancer of the rectum':ab,ti

#4 'cancer of the upper rectum':ab,ti

#5 'cancer, rectum':ab,ti

#6 'malignancies of the rectum':ab,ti

#7 'malignancy, rectum':ab,ti

#8 'rectal cancer':ab,ti

#9 'rectal carcinogenesis':ab,ti

#10 'rectal malignancies':ab,ti

#11 'rectal malignancy':ab,ti

#12 'rectum malignancy':ab,ti

#13 #1 OR #2 OR #3 OR #4 OR #5 OR #6 OR #7 OR #8 OR #9 OR #10 OR #11 OR #12

#14 'suture'/exp

#15 'open-surgery ligature loop':ab,ti

#16 'surgical suture':ab,ti

#17 'surgical suture, device':ab,ti

#18 'surgical suture, device (physical object)':ab,ti

#19 'surgical suture, nos':ab,ti

#20 'surgical sutures':ab,ti

#21 'surgical sutures (physical object)':ab,ti

#22 'suture (physical object)':ab,ti

#23 'suture - object':ab,ti

#24 'sutures':ab,ti

#25 #14 OR #15 OR #16 OR #17 OR #18 OR #19 OR #20 OR #21 OR #22 OR #23 OR #24

#26 'reinforcing':ab,ti

#27 #25 AND #26

#28 'anastomosis leakage'/exp

#29 'anastomotic leak':ab,ti

#30 'anastomotic leakage':ab,ti

#31 #28 OR #29 OR #30

#32 #13 AND #27 AND #31
