# Supplementary figures and images for: Efficacy of reinforcing sutures for prevention of anastomotic leakage after low anterior resection for rectal cancer: A systematic review and meta‐analysis
Source: Cancer Rep (Hoboken). 2024 Jan 4;7(2):e1941. doi: 10.1002/cnr2.1941 (PMC10849930; doi:10.1002/cnr2.1941)

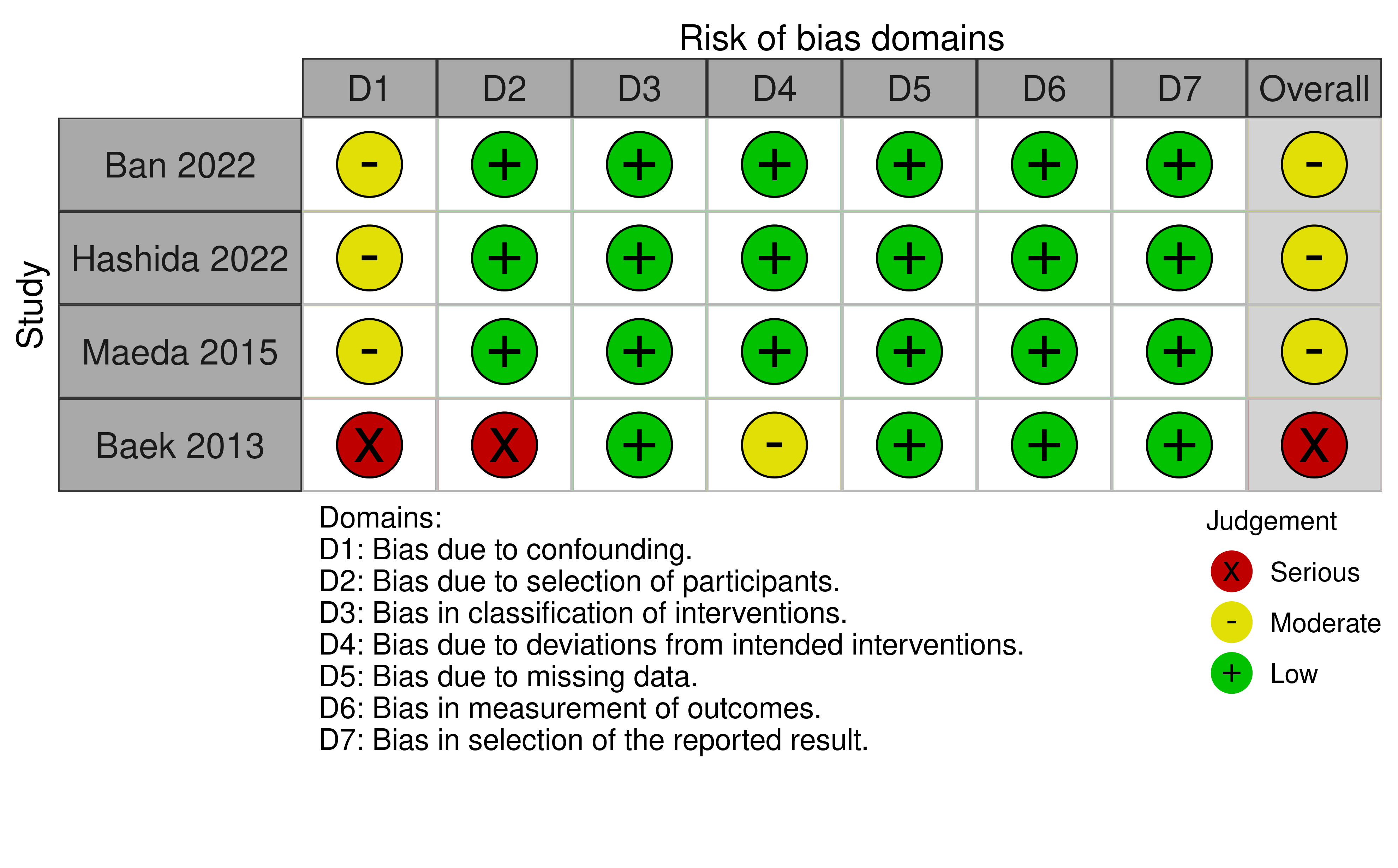

Supplement: Supplementary file 2 — Supplementary Material: Details of the risk of bias. [file CNR2-7-e1941-s003.zip › Risk of bias assessment for observational studies.tiff]

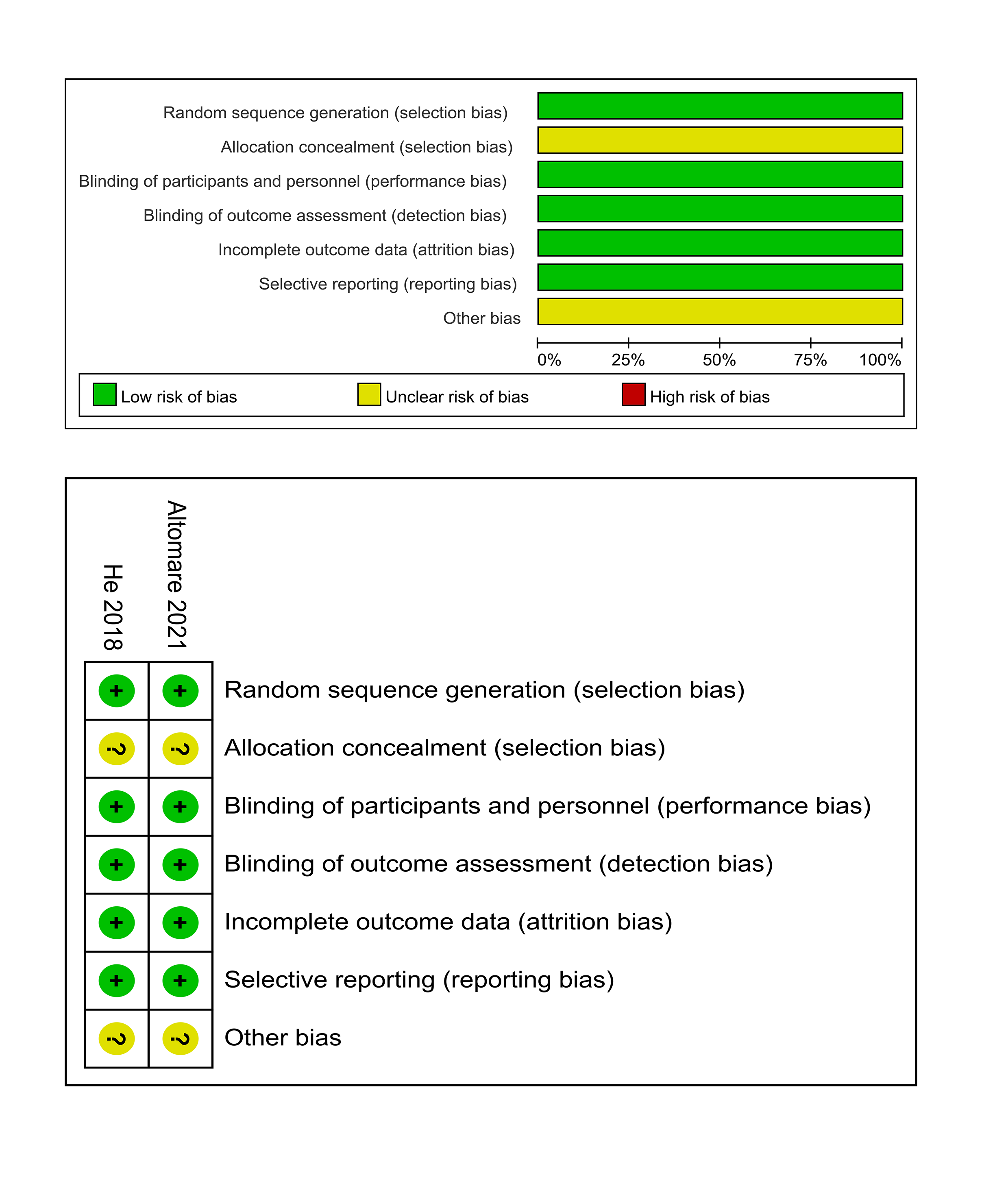

Supplement: Supplementary file 2 — Supplementary Material: Details of the risk of bias. [file CNR2-7-e1941-s003.zip › Risk of bias assessment for RCTs.tif]
