# Supplementary material for: Efficacy of reinforcing sutures for prevention of anastomotic leakage after low anterior resection for rectal cancer: A systematic review and meta‐analysis
Source: Cancer Rep (Hoboken). 2024 Jan 4;7(2):e1941. doi: 10.1002/cnr2.1941 (PMC10849930; doi:10.1002/cnr2.1941)
Supplement: Supplementary file 3 — Supplementary Material: GRADE assessments – anastomotic leakage. [file CNR2-7-e1941-s002.docx]

**GRADE Assessment**

| **Certainty assessment** | | | | | | | **№ of patients** | | **Effect** | | **Certainty** | **Importance** |
| --- | --- | --- | --- | --- | --- | --- | --- | --- | --- | --- | --- | --- |
| **№ of studies** | **Study design** | **Risk of bias** | **Inconsistency** | **Indirectness** | **Imprecision** | **Other considerations** | **Reinforcing sutures** | **Non-reinforcing sutures** | **Relative (95% CI)** | **Absolute (95% CI)** |  |  |
| **Anastomotic leakage (RCTs)** | | | | | | | | | | | | |
| 2 | randomised trials | not serious | not serious | serious^a^ | serious | none | 9/170 (5.3%) | 22/175 (12.6%) | **RR 0.50** (0.16 to 1.52) | **63 fewer per 1,000** (from 106 fewer to 65 more) | ⨁⨁◯◯ Low | CRITICAL |
| **Anastomotic leakeage (NRS)** | | | | | | | | | | | | |
| 4 | observational studies | serious^b^ | not serious | serious^a^ | not serious | none | 15/378 (4.0%) | 47/405 (11.6%) | **RR 0.37** (0.20 to 0.68) | **73 fewer per 1,000** (from 93 fewer to 37 fewer) | ⨁⨁◯◯ Low | CRITICAL |

**CI:** confidence interval; **RR:** risk ratio

#### Explanations

a. There was significant heterogeneity in suturing approach (transanal vs transabdominal), location of anastomosis, and use of diverting ileostomy between studies.

b. One study at serious RoB and showed different effect compared to other moderate RoB studies.
